# Limitations of caspofungin in the treatment of obstructive pyonephrosis due to *Candida glabrata *infection

**DOI:** 10.1186/1471-2334-6-126

**Published:** 2006-08-08

**Authors:** Silke Schelenz, Calum N Ross

**Affiliations:** 1 Microbiology Department, Norfolk and Norwich University Hospital, UK; 2 Department of Renal Medicine, Norfolk and Norwich University Hospital, UK

## Abstract

**Background:**

Caspofungin is a new antifungal agent with high-level activity against a number of *Candida *species including those that are resistant to azoles. Its good safety profile and low nephrotoxicity makes it an attractive drug to treat fungal infections in patients with compromised renal function. However, little is known about the clinical efficacy in the treatment of complicated urinary tract infections due to *Candida *species such as pyonephrosis.

**Case presentation:**

We report a case of obstructive pyonephrosis due to an azole (fluconazole and itraconazole) resistant *Candida glabrata *strain that failed to respond to intravenous treatment with caspofungin. A sustained clinical and microbiological response was only achieved after percutaneous drainage and instillation of amphotericin B deoxycholate into the renal pelvis in combination with intravenous liposomal amphotericin B.

**Conclusion:**

This case demonstrates the limitation of intravenous antifungal agents such as caspofungin as the sole treatment of an obstructive upper urinary tract infection due to *Candida *species. In order to achieve long term sustained cure from an obstructive pyonephrosis, pus and fungal balls should be drained and an anti-fungal agent such as amphotericin B deoxycholate instilled locally. The pharmacokinetics and role of caspofungin in the treatment of complicated *Candida *urinary tract infection is reviewed.

## Background

Pyonephrosis refers to infected purulent urine in an obstructed urinary collecting system such as the renal pelvices. Although bacteria cause the majority of these infections, *Candida *species such as *C. glabrata *have been reported to be involved in 5% of cases [1].

We describe a case of pyonephrosis complicated by candidaemia due to an azole (fluconazole and itraconazole) resistant *C. glabrata *that had not resolved after treatment with intravenous (iv) caspofungin. Caspofungin is a novel antifungal agent belonging to the class of echinocandins with good antifungal activity against *Candida *species including azole resistant strains [2]. It is recommended for the treatment of candidaemia and other serious invasive *Candida *infections [3]. The role of antifungal agents and the importance of percutaneous nephrostomy drainage in the conservative management of *Candida *pyonephrosis is discussed.

## Case presentation

A 71-year-old lady with type II diabetes mellitus, treated with insulin, presented in February 2004 with acute renal failure due to bilateral ureteric obstruction and hydronephrosis. Six months prior to this acute admission, the patient was treated empirically with fluconazole for a histologically proven necrotising *Candida *infection of the bladder. Her acute obstruction was managed by the insertion of bilateral nephrostomy catheters. On the day of the procedure she developed rigors, vomiting and right loin pain despite antibiotic cover with amoxicillin (500 mg tds). She was pyrexial (38.9°C), her inflammatory markers were increased (WBC 23.2 × 10^9^/l, neutrophils 21.8 × 10^9^/l, CRP 300 mg/l) and her renal function compromised (creatinine 333 μmol/l). The blood and nephrostomy urine cultures grew a non- albicans *Candida *species. The pre-morbid history suggested that the current non-albicans *Candida *isolate might be an azole-resistant strain and on the basis of her acute renal failure, the patient was commenced on empirical intravenous liposomal amphotericin B (AmBisome 1 mg/kg/od). The organism was subsequently identified as *C.glabrata *with reduced susceptibility to fluconazole and itraconazole MIC fluconazole 16.0 mg/l, itraconazole 1.0 mg/l) but susceptible to amphotericin B (MIC 1.0 mg/l), caspofungin (MIC 0.5 mg/l), voriconazole (MIC < 0.03 mg/l).

The patient was treated for four weeks with iv liposomal amphotericin B during which time her left nephrostomy tube was replaced by a ureteric stent. Four months later she was readmitted to the hospital for an elective right ureteric stent insertion but she developed a severe urosepsis (temperature 39.4°C, WBC 45.5 × 10^9^/l, CRP > 300 mg/l, creatinine 407 μmol/l) during the procedure which was abandoned. AmBisome (1 mg/kg/od) was given shortly after the procedure but did not prevent the need for intensive care support. Blood cultures and urostomy urine again grew the azole resistant *C.glabrata*. Due to the recurrence of *Candida *sepsis despite previous treatment with amphotericin B and because of her acute renal failure it was felt that a different class of anti-fungal agent with good activity against *C.glabrata *and minimal nephrotoxicity should be used. Amphotericin B was therefore stopped after three days and replaced by iv caspofungin (70 mg loading dose, 50 mg od). Caspofungin was continued for 12 days during which time her blood and catheter urine became sterile and her inflammatory markers and temperature normalised.

However, as the stenting of her right kidney was unsuccessful, attributed to multiple intra-renal strictures and irregularity of the urothelial outline, a percutaneous nephrostomy tube was inserted to allow long-term drainage. This time AmBisome was given one day prior to the procedure and continued for a total of 11 days during which time no sepsis occurred. Nevertheless, nephrostomy fluid analysis revealed large numbers of pus and yeast cells and again grew the azole resistant *C.glabrata*. Because of the persistent *C. glabrata *infection of the renal pelvices, despite treatment with iv liposomal amphotericin B followed by a course of caspofungin, it was felt that active local irrigation with amphotericin B was necessary to reduce the fungal load and to ensure that the antifungal agent achieved the site of infection. Five ml of conventional amphotericin B (Fungizone, 200 μg/ml) was instilled into the nephrostomy tube which was then clamped for 30–60 minutes at a time. This procedure was repeated four times daily for four days. A urostomy sample of urine taken 48 hours later continued to grow *C. glabrata *and treatment was therefore repeated for a further five days in combination with iv liposomal amphotericin B. Subsequently the urine sterilised and routine monthly urine cultures remained negative. The patient has had no further recurrence of the *Candida *infection during a 16 month follow up.

## Discussion

*C. glabrata *is an emerging pathogen and it is now the most common non-albicans species causing candidaemia [4]. This organism is increasingly resistant to the first line anti-fungal agent fluconazole which poses a challenge in the treatment of serious *Candida *infections [3,4]. The conventional treatment of azole resistant *Candida *strains often requires traditional agents such as amphotericin B, which can cause nephrotoxicity. Caspofungin belongs to a new group of antifungals, the echinocandins, which is highly active against azole resistant non-albicans *Candida *species. It demonstrates good activity in vitro against *C. glabrata *with MICs ranging between of 0.19–2 μg/ml although the drug might not always be fungicidal against this pathogen [2,5,6].

Clinical trials with caspofungin have shown good efficacy in the treatment of candidaemia, *Candida *oesophagitis and individual case reports have shown treatment successes with other serious *Candida *infections such as endocarditis [6-8]. The drug is well tolerated and, due to its principally hepatic excretion, it is safe to use in renal failure [6].

Very little is known about the clinical efficacy of caspofungin in the treatment of complicated fungal urinary tract infections such as pyonephrosis. Animal studies have shown that caspofungin can be detected in kidney tissue at concentrations three times that of plasma levels 24 hours after injection [9]. Similar pharmacokinetics were reported by *Stone et al*. who detected 4.2% of the original caspofungin dose in animal kidneys versus 35.2% in liver tissue after 24 hours [10]. Experiments in mice have also demonstrated that caspofungin can significantly reduce the *C.glabrata *tissue burden in kidneys at doses as low as 0.3 mg/kg/day although organ sterilization may only occur at much higher drug doses of 5 mg/kg/day or in combination with amphotericin B [5,11].

Unfortunately, the drug concentration in the kidney does not reflect availability in the urine. In healthy individuals, only a small fraction (1.44%) of the original drug can be detected after 24 hours in the urine after a single iv dose [10,12]. Caspofungin may therefore not be the drug of choice in the treatment of serious urinary tract infections although more clinical data is needed to fully assess the role of this drug for the treatment of such infections.

Another important issue in the management of pyonephrosis is the accumulation of pus and fungi in an obstructed space, the renal pelvices. As with other enclosed bacterial infections such as empyemas, drainage of the pus is of fundamental importance and may explain why systemic treatment with antifungals such as amphotericin B, caspofungin or fluconazole alone have proven to be unsuccessful [13,14].

In cases of pyonephrosis the insertion of a percutaneous nephrostomy tube allows urinary diversion with subsequent improvement in renal function and also enables the local administration of an anti-fungal drug directly to the site of infection [15]. Our case report supports the sparse literature suggesting that drainage via a percutaneous nephrostomy tube combined with the local instillation in combination with systemic use of an antifungal agent such as caspofungin or amphotericin B is necessary to successfully manage such an infection [13,14]. Other anti-fungal drugs such as flucytosine or voriconazole may also need to be considered.

## Conclusion

The intravenous anti-fungal drugs such as caspofungin or amphotericin B are useful in treating or preventing candidaemia during instrumentation such as insertion of nephrostomy tubes in cases of *Candida *pyonephrosis. However, they are unlikely to lead to a cure of this infection if used as the sole treatment. Conservative management should also include local drainage of pus and fungi as well as instillation of an effective antifungal agent into the pelvices.

## Abbreviations

MIC: minimal inhibitory concentration

## Competing interests

The author(s) declare that they have no competing interests.

## Authors' contributions

SS wrote the manuscript and was involved with the patients management. CNR cared for the patient and contributed to the writing of the manuscript. All authors read and approved the final manuscript.

## Pre-publication history

The pre-publication history for this paper can be accessed here:


